# Mass-spectrometric profiling of cerebrospinal fluid reveals metabolite biomarkers for CNS involvement in varicella zoster virus reactivation

**DOI:** 10.1186/s12974-017-1041-0

**Published:** 2018-01-17

**Authors:** Maike Kuhn, Kurt-Wolfram Sühs, Manas K. Akmatov, Frank Klawonn, Junxi Wang, Thomas Skripuletz, Volkhard Kaever, Martin Stangel, Frank Pessler

**Affiliations:** 1 TWINCORE Centre for Experimental and Clinical Infection Research GmbH, Feodor-Lynen-Str. 7, 30625 Hannover, Germany; 2 Helmholtz-Centre for Infection Research, Inhoffenstr. 7, 38124 Braunschweig, Germany; 3 Centre for Individualized Infection Medicine, Feodor-Lynen-Str. 15, 30625 Hannover, Germany; 4 Clinical Neuroimmunology and Neurochemistry, Department of Neurology, Hannover Medical School, Carl-Neuberg-Str. 1, 30625 Hannover, Germany; 5 Ostfalia University, Salzdahlumer Str. 46/48, 38302 Wolfenbüttel, Germany; 6 Research Core Unit Metabolomics, Hannover Medical School, Carl-Neuberg-Str. 1, 30625 Hannover, Germany; 7 Center for Systems Neuroscience, Bünteweg 2, 30559 Hannover, Germany

**Keywords:** Amino acid, Biomarker, Cerebrospinal fluid, CNS infections, Encephalitis, Herpes zoster, Metabolomics, Phosphatidylcholine, Sphingolipid, Varicella zoster virus

## Abstract

**Background:**

Varicella zoster virus (VZV) reactivation spans the spectrum from uncomplicated segmental herpes zoster to life-threatening disseminated CNS infection. Moreover, in the absence of a small animal model for this human pathogen, studies of pathogenesis at the organismal level depend on analysis of human biosamples. Changes in cerebrospinal fluid (CSF) metabolites may reflect critical aspects of host responses and end-organ damage in neuroinfection and neuroinflammation. We therefore applied a targeted metabolomics screen of CSF to three clinically distinct forms of VZV reactivation and infectious and non-infectious disease controls in order to identify biomarkers for CNS involvement in VZV reactivation.

**Methods:**

Metabolite profiles were determined by targeted liquid chromatography-mass spectrometry in CSF from patients with segmental zoster (shingles, *n* = 14), facial nerve zoster (*n* = 16), VZV meningitis/encephalitis (*n* = 15), enteroviral meningitis (*n* = 10), idiopathic Bell’s palsy (*n* = 11), and normal pressure hydrocephalus (*n* = 15).

**Results:**

Concentrations of 88 metabolites passing quality assessment clearly separated the three VZV reactivation forms from each other and from the non-infected samples. Internal cross-validation identified four metabolites (SM C16:1, glycine, lysoPC a C26:1, PC ae C34:0) that were particularly associated with VZV meningoencephalitis. SM(OH) C14:1 accurately distinguished facial nerve zoster from Bell’s palsy. Random forest construction revealed even more accurate classifiers (signatures comprising 2–4 metabolites) for most comparisons. Some of the most accurate biomarkers correlated only weakly with CSF leukocyte count, indicating that they do not merely reflect recruitment of inflammatory cells but, rather, specific pathophysiological mechanisms. Across all samples, only the sum of hexoses and the amino acids arginine, serine, and tryptophan correlated negatively with leukocyte count. Increased expression of the metabolites associated with VZV meningoencephalitis could be linked to processes relating to neuroinflammation/immune activation, neuronal signaling, and cell stress, turnover, and death (e.g., autophagy and apoptosis), suggesting that these metabolites might sense processes relating to end-organ damage.

**Conclusions:**

The results provide proof-of-concept for the value of CSF metabolites as (1) disease-associated signatures suggesting pathophysiological mechanisms, (2) degree and nature of neuroinflammation, and (3) biomarkers for diagnosis and risk stratification of VZV reactivation and, likely, neuroinfections due to other pathogens.

**Trial registration:**

Not applicable (non-interventional study).

**Electronic supplementary material:**

The online version of this article (10.1186/s12974-017-1041-0) contains supplementary material, which is available to authorized users.

## Background

Varicella zoster virus (VZV; termed human alphaherpesvirus 3 by the International Committee on Taxonomy of Viruses) is the causative agent of chicken pox. After this primary infection or immunization with attenuated VZV, latent viral reservoirs persist in neuronal ganglia along the neuraxis, and upon reactivation, the virus spreads along the nervous system. The lifetime risk of reactivation is 20–30% but increases with age or immunosuppression, presumably due to waning T cell immunity [1, 2]. Even though virostatic treatments are available, the clinical presentation of VZV reactivation spans the spectrum from uncomplicated herpes zoster (shingles), over involvement of cranial nerves, to a life-threatening meningoencephalitis. Complications include vasculitis, increased risk of myocardial infarction, or post-herpetic neuralgia, which affects up to 20% of all patients [3], and the overall mortality of VZV reactivation in the USA is nearly 5% [4]. Considering this wide spectrum of clinical severity and complications, there is a great need for biomarkers that would aid in early diagnosis, risk stratification, and outcome prediction and that would help to elucidate the mechanisms of clinical variability.

If VZV reactivation is suspected based on clinical examination, current diagnostics include serology, PCR, and culture. The value of VZV culture is limited due to the low sensitivity of around 45% [5]. VZV PCR has a high specificity (>95%) and sensitivity (80–95%) [6], but CSF viral load does not correlate well with outcome [7, 8]. Its usefulness may also be limited at early stages of disease, as PCR turns negative within about 1–3 weeks of detectable infection or reactivation. Antibodies are not detected up to two weeks after acute infection and can persist in humans after initial infection [9]. In terms of pathogen-independent CSF parameters, pleocytosis is usually present in VZV reactivation affecting the CNS [10] but can be absent especially in immunocompromised patients [11] or complications such as VZV vasculopathy [12]. With respect to experimental markers, increased CSF concentrations of neurofilament protein and glial fibrillary acidic protein were recently described in VZV-infected patients with facial palsy, indicating neuronal damage and astrogliosis, but their levels did not correlate with outcome [13, 14].

As demonstrated in recent studies on neurodegenerative disorders (e.g., Alzheimer’s disease, amyotrophic lateral sclerosis, and multiple sclerosis), metabolomic and/or lipidomic profiling of CSF has considerable potential for identifying biomarker candidates and suggesting disease-associated metabolic networks, also in the context of neuroinflammation [15–19]. The work on HIV-associated neurocognitive disorder revealed that CSF lipid profiles correlate with increasing severity of neurocognitive pathology in HIV-positive humans and simian immunodeficiency virus-infected macaques [20, 21], suggesting that CSF metabolomics could be successfully applied to neuropathology associated with an infectious disease. Despite these encouraging results and the well-documented observations that altered CNS glucose and lactate concentrations are associated with certain types of CNS infections, thus far, only three studies (all focusing on glucose metabolism) have applied small molecule profiling to discover CSF biomarkers for neuroinfections [22–24]. These studies used nuclear magnetic resonance spectroscopy and positron emission tomography/computed tomography and were therefore limited to the detection of relatively abundant analytes. A more recent study applied an untargeted LC-MS approach to analyze CSF in the context of the parasitic disease trypanosomiasis and reported 11 CSF metabolites to distinguish among disease stages [25].

The lack of a small animal model for VZV infection and reactivation has been impeding studies of its pathogenesis, meaning that such studies at the organismal level depend on analyses of biosamples from well-characterized patients or samples obtained at autopsy. The use of the latter is limited due to well-known artifacts from post-mortem changes. Considering (1) the successful applications of CSF metabolite profiling for biomarker discovery in the aforementioned studies and (2) the need for diagnostic and prognostic biomarkers for VZV reactivation and a better understanding of its pathogenesis including the nature of the associated neuroinflammation, we aimed to identify novel small-molecule CSF signatures and biomarkers for this multifaceted disorder.

## Methods

### Study population

CSF samples were obtained during initial routine lumbar puncture, were performed according to standard guidelines, and were collected prospectively in the context of the establishment of a CSF biobank encompassing a variety of neurological disorders. The patients whose samples were included in the present study were seen between 2005 and 2013 for routine neurological evaluation. The samples to be analyzed were selected retrospectively in 2015. This study was approved by the Ethics Committee of Hannover Medical School (file no. 2413-2014) and was conducted according to the Helsinki Declaration. Informed consent was waived due to the use of anonymized patient data. Inclusion criteria for evidence of VZV reactivation were either detection of VZV in CSF by PCR, intrathecal synthesis of VZV IgG, or the typical skin rash. Patients were categorized according to three different VZV reactivation patterns: (1) segmental zoster (Z. segmental, *n* = 14; defined by the presence of the typical cervical, thoracic, or lumbar rash); (2) facial nerve zoster (Z. facial, *n* = 16; facial nerve palsy with evidence of VZV infection, six of whom had the typical rash in the facial nerve distribution); and (3) zoster meningitis and/or encephalitis (Z. meningoencephalitis, *n* = 15; clinically confirmed CNS involvement such as altered mental status, focal neurological deficits, or meningeal signs plus evidence of VZV infection). Three control groups were included: (1) enteroviral meningitis (ent. men, *n* = 10; defined as clinically confirmed CNS involvement and detection of enteroviruses in CSF by PCR) as infectious disease control; (2) idiopathic facial paresis (Bell’s palsy, *n* = 11, defined as facial paralysis with normal CSF leukocyte count, excluding patients with evidence of infectious diseases, such as CSF PCR positive for VZV or HSV, or intrathecal specific antibody production for VZV, HSV, or *Borrelia burgdorferi* sensu *lato*); and (3) normal pressure hydrocephalus (controls, *n* = 15; defined as normal CSF pressure, cranial computed or magnetic resonance tomography scan indicative of normal pressure hydrocephalus, and at least one symptom of the Hakim triad [26, 27], excluding other symptoms as non-infected/non-inflamed CSF controls). Upon lumbar puncture, CSF samples were processed within 2 h to minimize time-dependent changes in CSF metabolomes. Aliquots of cell-free CSF were obtained by centrifugation and kept frozen until analysis for the present study. The following CSF parameters were analyzed directly after lumbar puncture: leukocyte count (counted manually with a Fuchs-Rosenthal counting chamber), protein concentration (Bradford dye-binding assay), lactate concentration, Q-albumin ratio (albumin concentration in CSF/albumin concentration in serum [28]), IgG-index (IgG concentration in CSF/IgG concentration in serum divided by Q-albumin ratio; age-adjusted reference limit = 4+(age/15) [28]), and PCR for viral diagnostics. IgG and albumin were measured in CSF and serum in the same latex-enhanced assay by kinetic nephelometry (Beckman Coulter IMMAGE). All methods are quality assured by participating in external quality control programs, the CSF survey of INSTAND [29]. A clinical epidemiological analysis, including the standard CSF parameters, of the same VZV reactivation patients is included in a separate manuscript (Skripuletz et al., manuscript submitted). Patients with VZV reactivation were treated with acyclovir (10 mg/kg body weight for 14 days, intravenously) as standard of care. Ten patients in the Z. facial group were treated with steroids (1 mg/kg body weight/day). Patients in the Bell’s palsy group were treated with steroids (1 mg/kg body weight/day) for 10 days with subsequent tapering. CSF was obtained prior to onset of acyclovir or steroid treatment.

### Metabolite profiling

We applied the AbsoluteIDQ®-p180 kit (Biocrates Life Science AG, Innsbruck, Austria) for CSF analysis [30] on a triple-quadrupole mass spectrometer (API4000, Sciex, Framingham, MA, USA) with an electrospray-ionization ion source coupled to a high-performance liquid chromatography system (SIL-HTc, Shimadzu, Japan). This combination allows to detect (1) 21 amino acids and 21 biogenic amines by liquid chromatography separation followed by targeted tandem mass spectrometry (MS/MS) and (2) 91 glycerophospholipids (phosphatidyl- and lysophosphatidylcholines) and isomers, 40 acylcarnitines, 15 sphingolipids (sphingo- and hydroxysphingomyelins) and isomers, and the sum of hexoses by direct infusion MS/MS (flow injection analysis). Thus, up to 188 metabolites can be analyzed per sample. All assays were performed according to the manufacturer’s recommendations (user manual UM_p180_ABSciex_11 and application note 1003-1, Biocrates Life Science AG, Innsbruck, Austria). In brief, manufacturer’s internal standard solution and calibrator/quality control samples or 30 μL of CSF were loaded onto a filter plate, derivatized using 5% phenylisothiocyanate in ethanol/water/pyridine (1/1/1, *v*/*v*/*v*), and extracted using 5 mM ammonium acetate in methanol. Remaining extracts were diluted and measured according to the manufacturer’s instructions. The following lipid nomenclature is used: sphingomyelin (SM) and hydroxysphingomyelin (SM(OH)): *Cx*:*y*: *x* = total number of carbon atoms and *y* = total number of double bonds in the amide bond. Phosphatidylcholines (PC): aa: both side chains are fatty acids linked to glycerol backbone by ester bonds, ae: one of the side chains is a fatty alcohol linked to glycerol backbone by an ether bond, and *Cx*:*y*: *x* = total number of carbon atoms and *y* = total number of double bonds in both fatty acid chains. Peak integration and calculation of metabolite concentrations were performed with the Analyst® (version 1.5.2, Sciex, Framingham, MA, USA) and MetIDQ™ software (Biocrates Life Science AG, Innsbruck, Austria) [30]. Metabolites with concentrations above the limit of detection (>LOD) were included in the subsequent statistical analyses.

### Statistical analyses

In order to generate a dataset that can be used for statistical analyses not compatible with missing values, values below LOD were replaced by multiple imputation using SPSS Statistics for Windows, version 20 (IBM Corporation, Armonk, NY, USA; from here on referred to as SPSS), according to the SPSS Missing Values Manual [31], using age, sex, diagnosis, and CSF leukocyte count as predictors. Data from five imputations were pooled to generate a “pooled estimate” dataset which was used for all analyses except jackknife cross-validation and random forest construction (see below). Standard statistical analyses were performed with the “stats” package of the R Foundation for Statistical Computing (version 3.2.3; from here on referred to as “R”) [32] or SPSS. Significance of between- and among-group differences was defined as a *P* value of < 0.05 unless stated otherwise. Corrections for multiple-hypothesis testing were not performed in those cases where data were shown to visualize the distribution of differential expression (Fig. 3) or correlations (Fig. 5), but a Benjamini-Hochberg correction [33] was applied when selecting specific markers (Fig. 4c–f). Nonmetric multidimensional scaling (MDS) of metabolite concentrations was done with the R package “MASS.” Spearman correlation analysis was used to assess correlations between leukocyte count and metabolite concentrations using SPSS.

The leave-one-out (jackknife) method was used for internal cross-validation, which was necessary because an external validation based on additional samples was not feasible due to the low incidences of some of the etiologies studied. Leave-one-out cross-validation is recommended as a validation strategy if no independent test dataset is available and sample sizes are limited [34, 35]. Briefly, the internal cross-validation process splits the cohort into a training subset (*n* = 80 patients) and a validation subset (*n* = 1 patient) and computes the best classifier on the training subset. The selected classifier is then tested on the validation subset. In an iterative approach, every sample is used as the validation subset and all classifiers are ranked according to the frequency of selection, with the most frequently selected classifiers constituting the best validated biomarkers. In detail, values <LOD were imputed with the *k*-nearest neighbors algorithm and the Heterogeneous Euclidean Overlap Metric (HEOM) [36]. Features were then selected based on the AUC values of the biomarker candidates on the training dataset, followed by the construction of a random forest classifier based on the 10 biomarker candidates with the highest AUC values. Subsequently, the classifier was tested on the single sample that was left out during the corresponding iteration of the jackknife method. In each iteration of this method, a different subset of biomarkers can be selected. In the end, a random forest classifier based on the biomarkers that were most often selected was again evaluated based on the jackknife method. Since this can lead to a selection bias [35], the procedure described above was repeated with 1000 bootstrap samples in order to compute a confidence interval (CI) for the AUC of the final classifier (i.e., small subset of metabolites).

## Results

### Study population

Table 1 summarizes sociodemographic and clinical features of the study population and results of standard diagnostic parameters. Consistent with the epidemiology of the diseases, median age was lower in the ent. men and Bell’s palsy groups. Peripheral blood leukocyte count and serum C-reactive protein (CRP) concentrations did not differ significantly across groups, although the highest individual CRP values were measured in Z. meningoencephalitis. CSF leukocyte count was two- to fourfold higher in patients with Z. facial and Z. meningoencephalitis than in the other groups, whereas total CSF protein concentration was lowest in controls. IgG index was lower in Bell’s palsy but was similar among the other groups. Lactate concentration was highest in Z. meningoencephalitis, and blood-CSF-barrier disruption was most pronounced in Z. meningoencephalitis, followed by ent. men and Z. facial. Taken together, these results demonstrate that the included patient groups (and CSF samples) have the features expected from their clinical diagnoses and that controls and idiopathic Bell’s palsy can be considered non-inflamed controls for the purpose of this study.

**Table 1:** Demographic and clinical laboratory characteristics

	Control (*n* = 15)	Bell’s (*n* = 11)	Ent. men (*n* = 10)	Z. segmental (*n* = 14)	Z. facial (*n* = 16)^a^	Z. men_enc (*n* = 15)^b^	*P* value^c^ (all patient groups)	*P* value^c^ (VZV-positive subgroups only)
Demographic								
Sex [%]								
Female	47	55	40	57	56	40	0.89^d^	0.57^d^
Male	53	45	60	43	44	60		
Age [years]								
Median (range)	64 (36–91)	45 (22–83)	32.5 (22–76)	60 (49–79)	64.5 (20–89)	55 (13–80)	*0.036*	0.634
Mean (SD)	64 (17)	46 (20)	41 (19)	61 (10)	59 (22)	53 (21)		
Blood parameter								
Leukocyte count [1000/μL]								
Median (range)	6.7 (1.0–11.5)	8.3 (4.6–11.9)	7.3 (4.0–14.0)	5.9 (2.4–9.0)	6.7 (4.1–13.2)	6.9 (3.8–13.6)	0.60	0.51
Mean (SD)	6.8 (2.6)	8.2 (2.3)	7.5 (3.0)	6.1 (2.1)	7.2 (2.3)	7.8 (3.3)		
C-reactive protein [mg/L]								
Median (range)	2.5 (1–29)	3 (1–31)	4 (1–39)	2 (1–15)	2.5 (1–18)	2 (1–128)	0.78	0.88
Mean (SD)	5.9 (9.2)	6.3 (9.6)	9.3 (12.3)	4.5 (4.4)	4.4 (5.0)	13.6 (32.3)		
CSF parameter								
Leukocyte count [1/μL]								
Median (range)	1 (0–11)	2 (0–5)	9 (1–619)	2 (0–18)	17 (1–800)	38 (2–1536)	*4.0E−06*	*7.0E−04*
Mean (SD)	1.9 (2.8)	2.1 (1.5)	129 (235)	3.3 (4.5)	156 (272)	200 (400)		
Protein concentration [mg/L]								
Median (range)	385 (255–656)	490.5 (309–829)	518 (240–976)	554.5 (273–880)	503 (270–1485)	649 (313–2048)	0.086	0.29
Mean (SD)	435 (134)	497 (156)	569 (205)	533 (139)	641 (342)	823 (486)		
IgG index								
Median (range)	0.51 (0.47–0.60)	0.47 (0.41–0.59)	0.53 (0.47–0.63)	0.54 (0.43–0.77)	0.54 (0.43–0.86)	0.55 (0.50–1.16)	*0.011*	0.65
Mean (SD)	0.53 (0.05)	0.48 (0.05)	0.54 (0.05)	0.56 (0.08)	0.57 (0.12)	0.63 (0.18)		
Lactate [mmol/L]								
Median (range)	1.64 (1.21–2.50)	1.57 (1.24–2.22)	1.88 (1.55–3.55)	1.78 (1.42–2.08)	1.87 (1.44–4.24)	2.73 (1.49–5.50)	*8.5E−04*	*0.006*
Mean (SD)	1.70 (0.30)	1.65 (0.34)	2.09 (0.62)	1.76 (0.18)	2.07 (0.67)	2.81 (1.18)		
Blood-CSF-barrier disruption [%]^e^								
No disruption	60	55	20	54	50	21	^f^	^f^
Light	40	45	70	39	31	36		
Moderate	0	0	10	7.7	19	29		
Severe	0	0	0	0	0	14		

*Bell’s* Bell’s palsy, *Ent. men* enteroviral meningitis, *men_enc* meningoencephalitis, *Z.* zoster

^a^This group comprised ten patients treated with steroids (1 mg/kg body weight per day) after CSF was obtained

^b^This group comprised five patients with meningitis, nine with encephalitis, and one with myelitis; these subgroups did not differ among each other in terms of the parameters in this table. Clinical complications included new cranial nerve paralysis and severe organic brain syndrome (*n* = 2 each). One patient received immunosuppressive medications at the time of lumbar puncture

^c^Calculated by Kruskal-Wallis test unless stated otherwise. Italic numbers: *P* ≤ 0.05, *P* values were not corrected for multiple-hypothesis testing

^d^Calculated by chi-square test

^e^Patient-adjusted *Q*-albumin reference value = 4 + (age/15). No disruption ≤ reference value, light ≤ 15, moderate ≤ 25, severe > 25

^f^Difference was significant only for Z. men_enc vs. control (*P* = 0.028, Fisher’s exact test)

### CSF metabolite detection

**Fig. 1:**
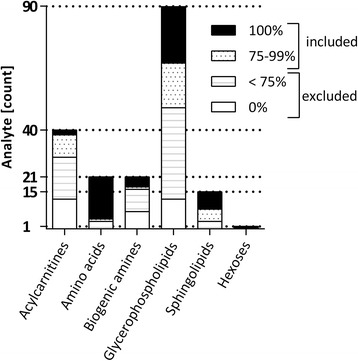
Detection efficiency of metabolites in CSF and selection for subsequent analyses. The number of detectable metabolites per analyte class is stated on the *y*-axis. Bars indicate the number of analytes in a given class detected >LOD in all samples according to four categories as indicated by the fill patterns: detected in all samples (“100%”), >75% of samples (“75–99%”), <75% of samples (“<75%”), and in none of the samples (“0%”). The 88 metabolites detected in 75–100% of all samples were used for subsequent analysis

All metabolites detected above LOD were initially considered and were grouped into four categories according to completeness of detection (Fig. 1). The groups “100%” (i.e., detected in 100% of samples; *n* = 54) and “75–99%” (*n* = 34) were merged and used for all subsequent analyses. The analytes detected in 75–99% and <75% of all samples did not comprise metabolites that were completely absent in any particular patient groups but present in the others, but were rather missing due to low abundance, or lack of detection due to technical reasons, across all groups. Nevertheless, the concentrations measured and the analytical coverage for the analyzed metabolites agreed with the information provided by the manufacturer [30] and the results published by Mandal et al. [37]. The subsequently included 88 analytes comprised 28% of acylcarnitines, 86% of amino acids, 24% of biogenic amines, 46% of glycerophospholipids, 80% of sphingolipids, and the sum of hexoses.

### Metabolite-based relationships among disease entities

**Fig. 2:**
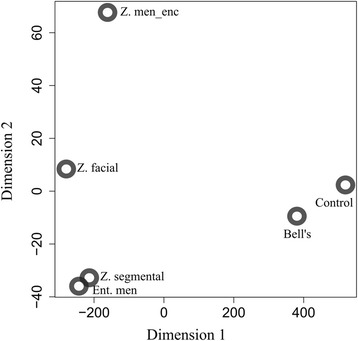
Recapitulation of the expected clinical relationships among the sample groups by CSF metabolite concentration profiles. Nonmetric multidimensional scaling (MDS) was performed using the pooled estimate dataset (see the “Methods” section) comprising the 88 metabolites that passed the quality screen (Fig. 1). Each circle represents the centroid of all samples in the respective group. The metabolite profiles separated the virally infected from the non-infected diagnoses along the first dimension and the three VZV reactivation forms according to increasing potential severity along the second dimension. The following abbreviations are used in all figures: *Bell’s* Bell’s palsy, *Ent. men* enteroviral meningitis, *men_enc* meningoencephalitis, *Z.* zoster

As a first proof of concept, we addressed the question whether the metabolite patterns would reflect the expected clinical relationships among the disease entities. Using MDS, the non-infected controls could be clearly separated from the virally infected groups along the first dimension, whereas the three forms of VZV reactivation could be separated along the second dimension in order of increasing potential severity and CNS involvement (Fig. 2). Interestingly, Z. segmental and ent. men clustered closely together, indicating that both sample groups are closely related based on their metabolite profiles. Likewise, Bell’s palsy and controls also clustered together, confirming their non-inflamed nature. These results were confirmed by Euclidean distance discriminatory analysis (data not shown). Thus, the metabolite patterns revealed clinically plausible differences and similarities among the sample groups.

### Global differences in metabolite profiles in VZV reactivation

**Fig. 3:**
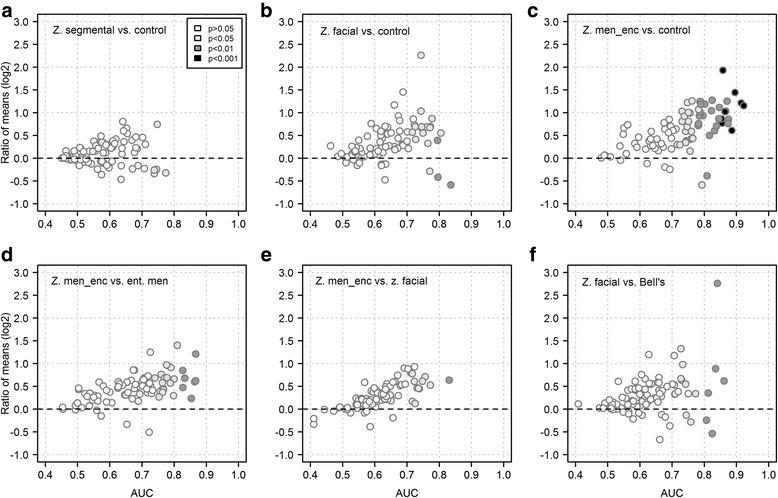
Extent of VZV reactivation correlates with the degree of reprogramming of CSF metabolite profiles. The group with the higher CSF leukocyte count constitutes the enumerator for calculating ratios of mean concentrations (log2, *y*-axis) and the positive state for calculating AUCs with binary ROC analysis (*x*-axis). Significance of AUCs was assessed by asymptotic uncorrected *P* values (expressed by fill color darkness as indicated in the legend within panel **a**) and by lower bound CI not crossing below 0.5. **a** Z. segmental vs. controls. Number of metabolites with AUC lower bound CI > 0.5 (CI > 0.5) = 7. **b** Z. facial vs. controls. (CI > 0.5) = 23. **c** Z. men_enc vs. controls. (CI > 0.5) = 56. **d** Z. men_enc vs. ent. men. (CI > 0.5) = 34. **e** Z. men_enc vs. Z. facial. (CI > 0.5) = 16. **f** Z. facial vs. Bell’s palsy. (CI > 0.5) = 18. The most pronounced and significant alterations in metabolite concentrations were observed in Z. men_enc, but reprogramming was also evident for Z. facial and, less so, Z. segmental

A combination of differential concentration analysis and binary ROC curve analysis was then used (1) to assess the degree of metabolite reprogramming in VZV reactivation and (2) to obtain a first overview of differences in concentration of the 88 metabolites within the clinically most relevant paired comparisons. We first tested whether CSF metabolite concentrations predominantly decreased or increased in the different disease states. Comparing the three forms of VZV reactivation combined against controls, concentrations of 27 metabolites (31%) were increased and only 3 metabolites (3.4%) were decreased (*P* < 0.05 in both cases), indicating that there was a tendency toward higher CSF metabolite concentrations in VZV infection. Regarding the individual VZV reactivation forms, there was a steady increase in the fraction of significantly (*P* < 0.05) elevated (with respect to controls) analytes from Z. segmental (*n* = 1; 3.4%) to Z. facial (*n* = 16; 18%) and to Z. meningoencephalitis (*n* = 50; 57%). In contrast, the fraction of downregulated analytes even decreased (Z. segmental: *n* = 4 [4.6%]; Z. facial: *n* = 3 [3.4%]; Z. meningoencephalitis: *n* = 2 [2.3%]). Of note, the downregulated analytes mostly comprised amino acids, with concentrations of serine being decreased in all three VZV reactivation forms. The discriminatory potential of the analytes was then assessed by the magnitude of the area under the ROC curve (AUC), the asymptotic *P* value of the ROC curve, and the ROC curve lower bound confidence interval (the number of analytes with lower CI > 0.5 for each comparison is shown in the legend to Fig. 3). The fraction of discriminatory markers increased according to the severity of VZV reactivation when comparing the three zoster presentations vs. controls (Fig. 3a–c). The degree of metabolic reprogramming in ent. men vs. controls was less pronounced than in Z. meningoencephalitis vs. controls (20 vs. 52 metabolites with *P* < 0.05), and consistent with these differences between the two viral etiologies, concentrations of 24 markers differed (*P* < 0.05) between Z. meningoencephalitis and ent. men (Fig. 3d). Differences between Z. meningoencephalitis and Z. facial were less pronounced (Fig. 3e). Of note, several metabolites differentiated also between Z. facial and idiopathic Bell’s palsy (Fig. 3f), two conditions that may have similar clinical features but cannot be discriminated easily on clinical grounds only. An overview about the regulation of significantly altered metabolites (uncorrected *P* value < 0.05, Kruskal-Wallis analysis across all groups) is shown in Additional file 1 (Figure S1) as a heatmap based on a hierarchical biclustering analysis. The patient group clustering analysis supports the findings shown in the nonmetric MDS plot (Fig. 2) but also indicates clades of similarly regulated metabolites, for instance a small group of downregulated amino acids.

### Forward selection and internal validation of biomarkers

**Fig. 4:**
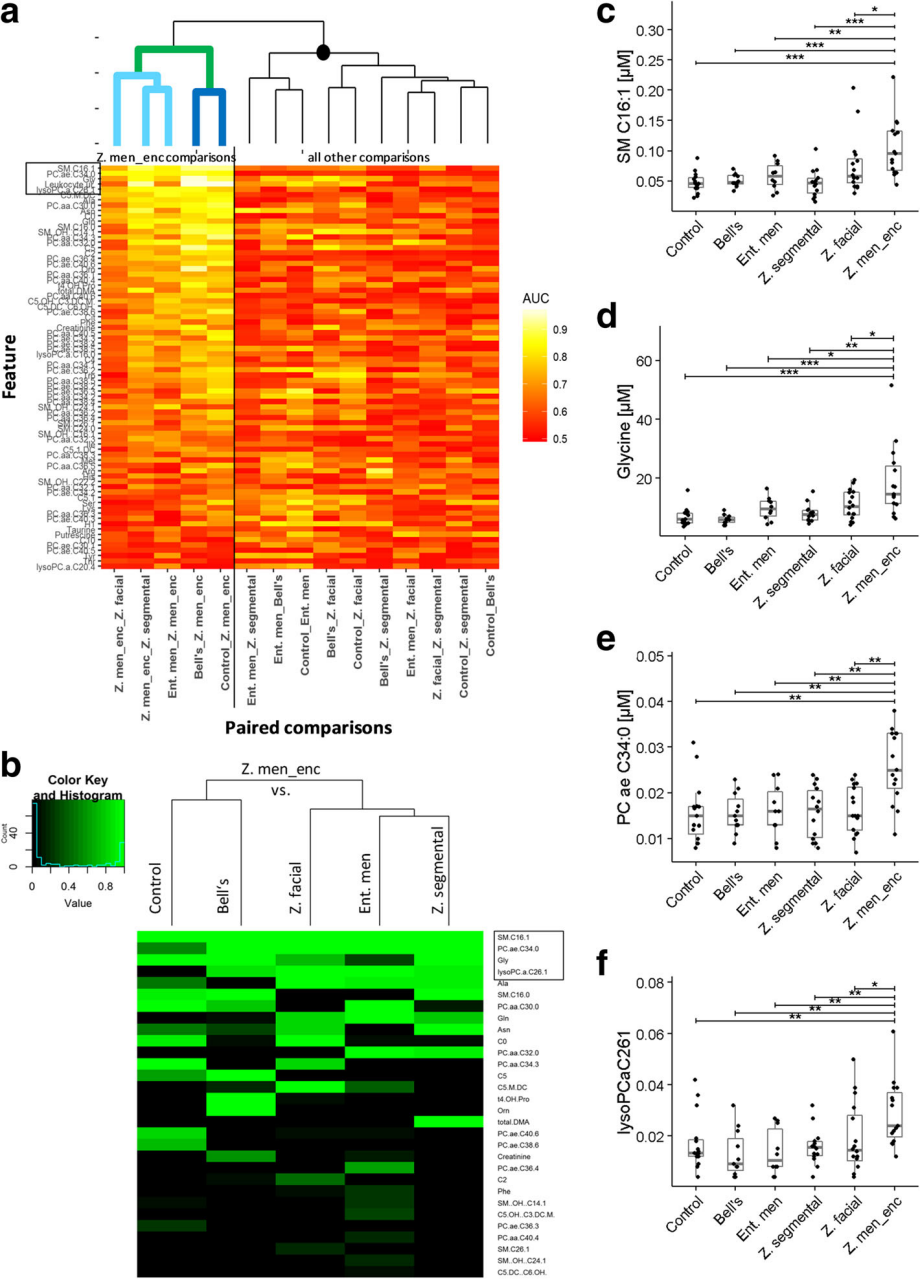
Selection and internal validation of biomarkers for Z. meningoencephalitis. **a** Hierarchical clustering analysis based on discriminatory ability of biomarker candidates. The number of features was reduced by including only the 60 analytes that were selected at least once by jackknife validation. The dendrogram was generated using the complete linkage method and AUCs (binary ROC analysis) of the selected analytes as input. The fill colors correspond to AUCs, as indicated in the legend within the panel. Analytes were ordered along the *y*-axis in descending order according to mean AUCs of the five possible paired comparisons involving Z. men_enc. Clade marked green: all comparisons featuring Z. men_enc. Subclade marked light blue: Z. men_enc vs. the virally infected groups. Subclade marked dark blue: Z. men_enc vs. the two non-inflamed control groups. The rectangle indicates the five best classifiers for Z. men_enc. The black circle identifies the clade containing the remaining comparisons involving the milder VZV reactivation forms. **b** Jackknife cross-validation. The dendrogram was constructed using the frequency with which a marker was selected (0 = never selected; 1 = always selected) among the 10 best classifiers for a given paired comparison. The rectangle identifies the four markers best validated for all paired comparisons. **c–f** Box/scatter plots depicting concentrations of the four biomarkers for Z. men_enc selected in Fig. 4b. Each dot represents one sample; the lower and upper hinges correspond to the first and third quartiles, respectively; the whiskers indicate 1.5 times the interquartile range to the lowest and highest value, respectively; horizontal lines indicate the median. Significance of concentration differences across all sample groups was determined with Kruskal-Wallis analysis (Benjamini-Hochberg correction for multiple-hypothesis testing, *P* = 0.014 as significance cutoff). **c** SM C16:1; *P* = 0.00114. **d** Glycine; *P* = 0.000568. **e** PC ae C34:0; *P* = 0.00568. **f** lysoPC a C26:1; *P* = 0.00852. Significant between-group differences are shown in the panels (Mann-Whitney *U* test, uncorrected *P* values, *< 0.05, **< 0.01, ***< 0.001)

The AUCs of the 60 biomarkers that were selected for a given diagnosis at least once in the jackknife cross-validation, as well as the AUC of leukocyte count, were used as input for a hierarchical clustering analysis in order to test whether these markers could define phylogenetic relationships specific for Z. meningoencephalitis (Fig. 4a). Indeed, all paired comparisons featuring Z. meningoencephalitis were contained in a single clade (marked green), which could be further divided into one clade containing the three virus-associated groups (light blue) and another clade containing the two non-inflamed control groups (dark blue). Of note, the five best markers for Z. meningoencephalitis comprised four metabolites belonging to three different analyte classes (glycine, SM C16:1, PC ae C34:0, and lysoPC a C26:1) and leukocyte count. The other main node of the dendrogram (black circle) divided into one clade containing three of the four remaining comparisons featuring ent. men, and a second more heterogeneous clade containing the remaining Z. facial and Z. segmental comparisons.

Jackknife internal cross-validation (see the “Methods” section) was then used to identify the best internally validated biomarker candidates for the comparisons between Z. meningoencephalitis and each of the other five groups (Fig. 4b). This analysis identified the same four best analytes as the AUC-based hierarchical clustering (Fig. 4a), suggesting that increased concentrations of these four metabolites were most closely associated with a diagnosis of Z. meningoencephalitis. As shown in Fig. 4c–f, concentrations of all four were highest in Z. meningoencephalitis, and concentrations were significantly lower (*P* < 0.05) in all other disease etiologies, even in ent. men, which was included as disease control (viral CNS infection of different etiology). When the cross-validation was applied to identify markers for the two milder forms of VZV reactivation in clinically relevant comparisons, there was more heterogeneity among the best validated markers for the various paired comparisons. Nonetheless, markers of moderate to excellent discriminatory ability could be validated for all comparisons (see below).

### Comparison of diagnostic performance of CSF metabolites and standard CSF parameters

Table 2 summarizes the results of ROC analysis for standard diagnostic parameters (section A), the best internally validated metabolite biomarkers for the most relevant comparisons involving the three VZV reactivation patterns (section B), and best classifiers (biomarker combinations, section C). Leukocyte count differentiated Z. meningoencephalitis accurately from Z. segmental, non-inflamed controls, and Bell’s palsy, but not from Z. facial or ent. men. The discriminatory ability of lactate came close to that of leukocyte count, whereas protein concentration had only low discriminatory value. The metabolite markers (section B) were inferior only in the two cases where leukocyte count already demonstrated high discrimination, but they showed significantly better discrimination where traditional markers had only limited value (e.g., Z. facial vs. Bell’s palsy, or Z. meningoencephalitis vs. Z. facial or ent. men). Lastly, optimal combinations of biomarkers (including leukocyte count, which was included because it is easily and rapidly determined in clinical practice) were selected by random forest construction (section C). This led to superior AUCs in all except two comparisons.

**Table 2:** Diagnostic value of standard CSF parameters, metabolites, and their combinations

	Z. men_enc vs.					Z. facial vs.			Z. segmental vs.	
	Control	Z. segmental	Z. facial	Ent. men	Bell’s	Z. segmental	Bell’s	Control	Bell’s	Control
(A) Standard CSF parameter										
Leukocyte count	0.97***	0.94***	0.63	0.68	0.96***	0.70	*0.72*	0.80*	0.54	0.68
Protein concentration	0.81*	0.67	0.62	0.62	*0.73*	0.52	0.59	0.69	0.61	0.68
IgG index	0.74*	0.62	0.60	0.66	0.90**	0.51	0.77*	0.58	0.83**	0.63
Lactate	0.86**	0.82**	0.71	0.70	0.88**	0.67	0.75*	0.74*	0.65	0.65
(B) Metabolites										
Best internally validated marker^a^	SM C16:1	SM C16:1	PC ae C34.0	lysoPC a C26:1	Glycine	PC aa C32:0	SM(OH) C14:1	Tryptophan	Arginine	Serine
AUC	0.92***	0.90***	0.83**	0.87**	0.96***	0.76*	0.86**	0.84**	0.90***	0.77*
(C) Best classifier^b^										
No. of markers	2	3	1	2	2	2	3	4	1	3
Markers (frequency)	Glycine (1.0) PC aa C30:0 (0.9)	Leukocytes (1.0) SM C16:1 (1.0) total DMA (0.60)	PC ae C34:0 (1.0)	lysoPC a C26:1 (1.0) PC aa C32:0 (1.0)	Leukocytes (1.0) Glycine (1.0)	Leukocytes (0.73) PC aa C32:1 (1.0)	SM(OH) C14:1 (1.0) Glycine (0.96) Tryptophan (1.0)	Tryptophan (1.0) SM(OH) C14:1 (0.71) Leukocytes (1.0) Serine (0.84)	Arginine (1.0)	Serine (1.0) PC ae C36:3 (0.88) Methionine (0.67)
AUC	1.00	0.95	0.83	0.90	1.00	0.77	0.92	0.86	0.90	0.86
95% CI	0.87–1.0	0.76–1.0	N/A	0.57–1.0	0.89–1.0	0.51–0.91	0.61–0.98	0.55–0.98	N/A	0.53–0.96

Values correspond to areas under the receiver operator characteristic (ROC) curve (AUC) performed on continuous variables. Asymptotic significance (not corrected for multiple-hypothesis testing): *P ≤ 0.05, **P ≤ 0.01, ***P ≤ 0.001. Italic values: lower CI > 0.5

*Bell’s* Bell’s palsy, *CI* confidence interval, *DMA* dimethylarginine, *Ent. men* enteroviral meningitis, *N/A* not applicable, *PC* phosphatidylcholine, *SM* sphingomyelin, *SM (OH)* hydroxysphingomyelin, *Z.* zoster, *Z. men_enc* Z. meningoencephalitis

^a^According to the frequency of selection (0 = never selected; 1 = always selected) in the leave-one-out cross-validation. The marker with the higher AUC was selected if two markers had the same frequencies

^b^Biomarker combination with the highest discriminatory ability identified by random forest construction as outlined in Methods. CIs of AUCs were evaluated based on 1000 bootstrap samples of the same size as the original data drawn with replacement

### Correlation between metabolite concentrations and leukocyte count

**Fig. 5:**
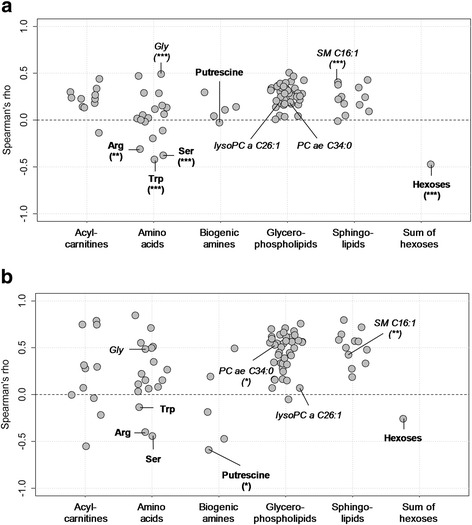
Spearman correlation between metabolite concentrations and leukocyte count in CSF by analyte group. **a** All patient groups. **b** Z. meningoencephalitis only. Values correspond to Spearman’s correlation coefficients (*ρ*). Analytes with significant negative Spearman’s *ρ* in one of the two correlation analyses are labeled bold and the four internally best validated biomarker candidates (Fig. 4c-f) in italics. Uncorrected *P* values: *P < 0.05; **P < 0.01; ***P < 0.001. Abbreviations: *Arg* arginine, *Gly* glycine, *Ser* serine, *Trp* tryptophan

CSF leukocyte count (“cell count”) is a currently used indicator for the degree of overall CNS inflammation, independent of underlying disease etiology, but is also used to aid in making important clinical decisions such as institution or termination of anti-infective medications. In order to test whether the four metabolites specific for Z. meningoencephalitis merely reflect the degree of CNS inflammation, as does CSF leukocyte count, or whether they are indicative of distinct pathophysiological mechanisms, we performed a Spearman’s correlation analysis between metabolite concentrations and CSF leukocyte count. Correlation with leukocyte count was not preferentially associated with any of the analyte classes (Fisher’s exact test, *P* = 0.18, *χ*^2^ = 7.181). Overall, there were weak-to-moderate correlations between analyte concentrations and leukocyte count, as indicated by comparatively low Spearman coefficients (Spearman’s *ρ* between − 0.5 and 0.5, Fig. 5a). Most correlations were positive, but amino acids formed a distinct exception in that there were also significant negative correlations. The three amino acids with significant negative Spearman’s *ρ* (*P* < 0.05, identified by labels) and the sum of hexoses were also among the analytes with decreased concentrations in VZV reactivation identified in Fig. 3. Of note, the Z. meningoencephalitis-associated metabolites selected by the cross-validation shown in Fig. 4 (SM C16:1, glycine, PC ae 34:0, and lysoPC a C26:1, marked in the figure) did not always correlate most strongly with leukocyte count. When limiting this analysis to Z. meningoencephalitis (Fig. 5b), the overall degree of correlation increased, but again, the four biomarker candidates were not among the metabolites with the strongest correlations. Thus, the observed differences in metabolite profiles among the sample groups did not merely reflect the degree of overall CNS inflammation, but other, presumably disease- or pathogen-specific, pathophysiological processes.

### Diagnostic comparison of leukocyte count and the four Z. meningoencephalitis-associated metabolites

To test their performance in a clinically relevant scenario, we then used standard measures of diagnostic test evaluation to assess the abilities of the four best validated metabolites (SM C16:1, glycine, PC ae 34:0, and lysoPC a C26:1) to diagnose Z. meningoencephalitis among all VZV-positive samples (Table 3). Leukocyte count was evaluated for comparison, as our data revealed it as the most accurate standard diagnostic marker (please refer to Table 2). Although it had some diagnostic value, the four metabolites performed equally well or better in most aspects, indicating their value as novel biomarkers for clinical application and as sensors of the associated neuroinflammation. In particular, lysoPC a C26:1 had by far the highest sensitivity and negative predictive value, and PC ae 34:0 had both perfect sensitivity and positive predictive value. These results also agree with our finding that dysregulation of these four metabolites in CSF did not correlate strongly with leukocyte count (Fig. 5). Both findings indicate that the identification of novel metabolite biomarker candidates by metabolite profiling of CSF has high potential to reveal biomarkers to supplement routine diagnostics.

**Table 3:** Comparison of leukocyte count against metabolite biomarkers to detect CNS infection in VZV reactivation

	Sensitivity	Specificity	PPV	NPV	AUC (95% CI)	*P* value^a^
Leukocyte count	0.80	0.70	0.57	0.88	0.77 (0.64–0.91)	0.003
SM C16:1	0.73	0.77	0.61	0.85	0.81 (9.68–0.94)	0.001
Glycine	0.80	0.70	0.57	0.88	0.78 (0.64–0.93)	0.002
lysoPC a C26:1	0.93	0.62	0.56	0.95	0.79 (0.66–0.93)	0.002
PC ae C34:0	0.53	1.00	1.00	0.80	0.84 (0.71–0.97)	< 0.0001

Values are based on binary comparison of Z. meningoencephalitis vs. the pool of Z. facial and Z. segmental. The metabolite biomarkers correspond to the four best internally validated metabolites (Fig. 4, Table 2)

*AUC* area under the receiver operating characteristic curve, *CI* confidence interval, *PPV* positive predictive value, *NPV* negative predictive value, *PC* phosphatidylcholine, *SM* sphingomyelin

^a^Uncorrected asymptotic *P* values

## Discussion

Using targeted mass spectrometric analysis, we identified metabolic signatures and small-molecule CSF biomarker candidates for three forms of VZV reactivation of differing extent of neuroinflammation and CNS involvement and evaluated these for the differentiation from non-inflamed controls and from ent. men as a viral CNS infection of different etiology.

### CSF metabolites associated with Z. meningoencephalitis

Elevated CSF concentrations of four analytes (glycine, SM C16:1, PC ae C34:0, and lysoPC a C26:1) were most closely associated with Z. meningoencephalitis, suggesting that they may represent potential biomarkers for this diagnosis and also reflect aspects of its pathogenesis. Their concentrations did not correlate directly with CSF leukocyte count, indicating that they do not merely reflect CNS inflammation but also pathological processes in CNS parenchymal cells including neurons. These markers belong to three different molecular classes, suggesting that VZV reactivation with CNS involvement does not preferentially affect a particular metabolic pathway. The association with Z. meningoencephalitis was strongest for SM C16:1. Sphingomyelins are integral components of cell membranes, including neuronal membranes, and their levels are regulated partially through a dynamic exchange with ceramides. Dysregulated SM levels are seen in processes associated with cell stress and death (e.g., autophagy and apoptosis) [38, 39] and in neurodegenerative diseases [18]. In particular, induction of autophagy due to endoplasmic reticulum stress is a hallmark of VZV infection and is believed to enhance viral spread due to prolonged survival of infected cells [40, 41]. SM C16:1 has been associated with inflammatory conditions in that elevated concentrations have been measured in serum from patients with sepsis [42]. In contrast, it was decreased in septic shock [42] and in serum of individuals with HIV-1 infections, i.e., two conditions associated with immune compromise [43]. Phosphatidylcholines (PC) play important roles in membrane-mediated cell signaling. While PC ae C34:0 has not been implicated in human infections, it has recently been shown that positive-strand RNA plant viruses stimulate PC synthesis at the site of viral replication [44]. It remains to be studied whether this also occurs in DNA virus infections and in humans. LysoPCs are normally minor constituents of cell membranes, but their abundance can potentially increase as they are derived from PCs by hydrolytic removal of a fatty acid group. When released from apoptotic cells, they can act as chemoattractants for macrophages [45], which can be potentiated by reactive oxygen species [46]. As SM C16:1, serum concentrations of lysoPC a C26:1 are elevated in sepsis [42], but its role in CSF and neuroinfections has not been studied. Glycine was detected at low concentrations but nonetheless turned out to be one of the most robust markers for Z. meningoencephalitis. Apart from its well-known metabolic functions as an amino acid, it is released by glycinergic neurons in the CNS (where it can act as an inhibitory neurotransmitter), it functions as an immunomodulatory immune effector and as a cytoprotective agent (reviewed in [47]). However, studies in rats have suggested that elevated CNS glycine concentrations lead to pathologic changes such as induction of reactive oxygen species and glial reactivity [48]. Therefore, the elevated glycine concentrations in Z. meningoencephalitis may have diverse implications for pathogenesis. Taken together, the above observations suggest that the close association of elevated levels of these four analytes with Z. meningoencephalitis is due to a combination of parenchymal cell stress, cell death, and the host immune response in the CNS. Further research is required to elucidate whether the metabolites play roles as effector molecules or whether they are predominantly the by-products of cellular demise. The amino acids arginine, tryptophan, and serine represented a special case in that their CSF concentrations correlated negatively with CSF leukocyte count. Possible explanations would be that their levels decrease in stressed or dying cells before they are released or that they are preferentially taken up and metabolized by inflammatory cells or stressed parenchymal cells.

### Are the identified markers specific for VZV?

From the point of view of biomarker research, it would be desirable to identify markers that are specific for selected pathogens or pathogen families. In the presented study, we used ent. men as a viral CNS disease control. Of note, of the four metabolites most closely associated with Z. meningoencephalitis, lysoPC a C26:1 was a highly accurate marker to differentiate Z. meningoencephalitis also from ent. men, and only glycine was among the 22 metabolites with significantly different concentrations (*P* < 0.05, lower CI > 0.5) between ent. men and controls and, in addition, it had low discriminatory potential (AUC = 0.74, rank 21/22). Preliminary analyses of samples from patients with HSV CNS infection (*n* = 9) have revealed a different set of metabolite markers specific for this diagnosis (Kuhn et al. unpublished data). Taken together, these results do suggest the possibility that the above identified four metabolites may be specific for VZV CNS infection. Pathogen-specific CSF metabolite signatures may result from differences in host-pathogen interactions such as viral tropism, type of infection (persistent vs. lytic), host immune responses, or blood-CSF-barrier function. On the other hand, differences that non-specifically reflect severity of infection and extent of tissue damage may blur, for instance, in cases of mild infections with normally highly pathogenic viruses (e.g., mild HSV meningitis) compared to severe cases of infection with a virus of normally relatively low virulence (e.g., severe enteroviral meningitis in an immunocompromised host). Clearly, additional cohorts also including different viral etiologies now need to be studied in order to address the above question more conclusively, and we are currently collecting samples for an external validation cohort.

### CSF metabolites associated with segmental and facial nerve VZV reactivation

Even though the results were not as clear cut as in the case of Z. meningoencephalitis, robust metabolite markers for the two focal forms of VZV reactivation were also identified (Table 2), and the MDS analysis clearly showed that the overall metabolite patterns differentiated Z. facial and Z. segmental not only from each other but also from the non-inflamed controls. Among the best metabolite markers for Z. facial were SM(OH) C14:1, tryptophan, creatinine, and PC aa C32:0, whereas Z. segmental-specific markers were mainly the amino acids methionine, arginine, histidine, and serine and also the acylcarnitine C5 and the sum of hexoses (results combined from best classifier and frequency analysis). It remains to be explained why altered amino acid concentrations were particularly associated with Z. segmental, but their overrepresentation is consistent with the notion that its pathogenesis differs from that of Z. meningoencephalitis. Highly discriminatory biomarkers were also identified even for the differentiation between Z. facial and idiopathic Bell’s palsy (SM(OH) C14:1, glycine, and tryptophan), two conditions that can present with the same clinical signs and symptoms. Infectious or inflammatory etiologies have been postulated for Bell’s palsy, but our results suggest that this is not the case, at least in individuals with normal CSF leukocyte count.

### Comparison of standard diagnostic markers and metabolite biomarker candidates

This is, to our knowledge, the first study to quantify the discriminatory ability of standard CSF parameters for the differentiation among distinct forms of VZV reactivation and diverse control groups. The results underscore the value of determining CSF leukocyte count in selected scenarios, for instance to evaluate the likelihood of disseminated CNS VZV infection. Elevated lactate concentrations (> 3.5 mM) are useful to differentiate bacterial from viral meningitis [49], but lactate also demonstrated considerable value in selected comparisons, particularly involving Z. meningoencephalitis. However, there were several scenarios where traditional CSF parameters were of limited value, for instance for differentiating between Z. meningoencephalitis and ent. men or Z. facial. Of note, metabolite biomarkers proved to be more accurate for these potentially clinically relevant comparisons, underscoring the potential importance of our findings to clinical practice. In addition, metabolite biomarkers might be particularly valuable in settings where established markers such as leukocyte count are unreliable, as in immunocompromised patients [50]. Selected combinations of markers led to further improvement of discrimination in all comparisons but one. All these examples imply that metabolomics screening of CSF can reveal metabolites superior to routine diagnostics for selected scenarios and therefore constitutes a powerful approach to identify novel biomarkers for CNS infections and other neurological diseases. Although CSF is not an easily accessible body fluid and lumbar puncture is an invasive method, it is nevertheless routinely performed and important to diagnose many diseases of the nervous system, as also emphasized by Reiber [28] and Zunt and Marra [51], among others. In addition, an increasing number of publications investigate CSF metabolite profiles especially for neurodegenerative disorders. We are currently preparing a study to assess the value of serum and CSF pairs for routine diagnostics in order to test whether peripheral blood metabolite markers might potentially replace CSF markers obtained by the invasion lumbar puncture. Nevertheless, the analysis of CSF is still the method of choice to diagnose CNS diseases. This primarily diagnostic study was not powered to assess associations between biomarker concentrations and disease severity within a given group. As outlined above, the metabolites most strongly associated with Z. meningoencephalitis could be functionally linked to CNS stress and, thus, end-organ damage of the infection. Considering that traditional diagnostics (including VZV viral load) do not predict disease severity or outcome, it is conceivable that CSF metabolites may prove superior for the early identification of patients requiring higher levels of care or follow-up. Along the same lines, it is possible that changes in CSF metabolites follow different kinetics than viral nucleic acids in CSF, allowing for the use of metabolite biomarkers outside the narrow time window during which PCR is positive.

### Limitations

Even though we used a relatively broad screen encompassing several analyte classes, the 88 analytes that passed the quality assessment represent only a subpopulation of potentially important molecules in CSF. Therefore, broader and more sensitive screens might unveil additional important metabolite changes. The annotated sphingo- and glycerophospholipids measured with the targeted MS/MS approach inherent to the Biocrates AbsoluteIDQ®-p180 kit can also stem from isomers and isobars [52], which makes it more difficult to infer about pathophysiological mechanisms. However, detection of a combined lipid signal can nevertheless be useful in biomarker research and diagnostics. As apparent from Fig. 1, 34 metabolites were detected only in 75–99% of all samples, which is inherent to the overall low abundance of these metabolites in CSF, but not to the absence or presence of these metabolites in any particular patient group. Nevertheless, our overall detection rate of 88/188 metabolites (=47%) agrees well with previous reports analyzing metabolites in CSF with these kits [30, 37]. Regarding sample quality, the samples were collected during an 8-year period, potentially introducing artifacts from differences in stability during storage. However, we did not find any obvious associations between analyte concentrations and year of sample collection (data not shown). We used samples from patients with normal pressure hydrocephalus as non-inflamed controls. Even though CSF from this condition is likely not completely comparable to CSF from healthy individuals, the standard CSF indices were within the normal range. CSF parameters may change with age, and we are not aware of any data regarding age-associated changes in CSF of the metabolites investigated. Only limited follow-up information was available from most patients, thus limiting our ability to include patient outcomes in the analyses. The limited sample numbers are inherent to the low incidences of some of the clinical presentations studied and the fact that lumbar puncture is only rarely performed on patients with uncomplicated shingles. We therefore used an internal cross-validation method to identify the most valid biomarker candidates.

## Conclusions

The results suggest that assessing CSF metabolite patterns may be a powerful tool for identifying small-molecule biomarkers for diagnosis and risk stratification of CNS infections, extent and nature of neuroinflammation, and to improve our understanding of the pathogenesis of VZV reactivation at the organismal level. Considering the observed differences between Z. meningoencephalitis and ent. men, it is tempting to speculate that patterns characteristic of certain pathogens or pathogen families can be identified which could aid in establishing a diagnosis in patients in whom a pathogen cannot be detected.

## Additional file

Additional file 1: Figure S1. Biclustering analysis based on differentially abundant metabolites. Fold changes (ratio of mean concentrations in each group relative to control) of metabolites with significant across-group differences (uncorrected Kruskal-Wallis *P* value < 0.05, *n* = 39) were used as input using the R function gplots::heatmap.2 (www.r-project.org, Authors: Andy Liaw; revised by R. Gentleman, M. Maechler, W. Huber, G. Warnes). Between-group relationships support those identified in the nonmetric MDS analysis (Fig. 2). The greatest concentration changes are evident in Z. meningoencephalitis. Some co-regulation of metabolites is evident in the dendrogram, in particular clustering of five almost exclusively downregulated metabolites (including Arg, Trp and Ser, and the sum of hexoses (H1), all of which correlated negatively with CSF leukocyte count, see Fig. 5). The apparent upregulation of C5 in the Z. facial group was due to four patients with high concentrations of unknown significance. (TIFF 337 kb)

## Abbreviations

AUC: Area under the curve
CI: Confidence interval
CRP: C-reactive protein
CSF: Cerebrospinal fluid
LOD: Limit of detection
MDS: Multidimensional scaling
MS/MS: Tandem mass spectrometry
PC: Phosphatidylcholine
ROC: Receiver operating characteristic
SM: Sphingomyelin
VZV: Varicella zoster virus
