# Supplementary figures and images for: Mass-spectrometric profiling of cerebrospinal fluid reveals metabolite biomarkers for CNS involvement in varicella zoster virus reactivation
Source: J Neuroinflammation. 2018 Jan 17;15:20. doi: 10.1186/s12974-017-1041-0 (PMC5773076; doi:10.1186/s12974-017-1041-0)

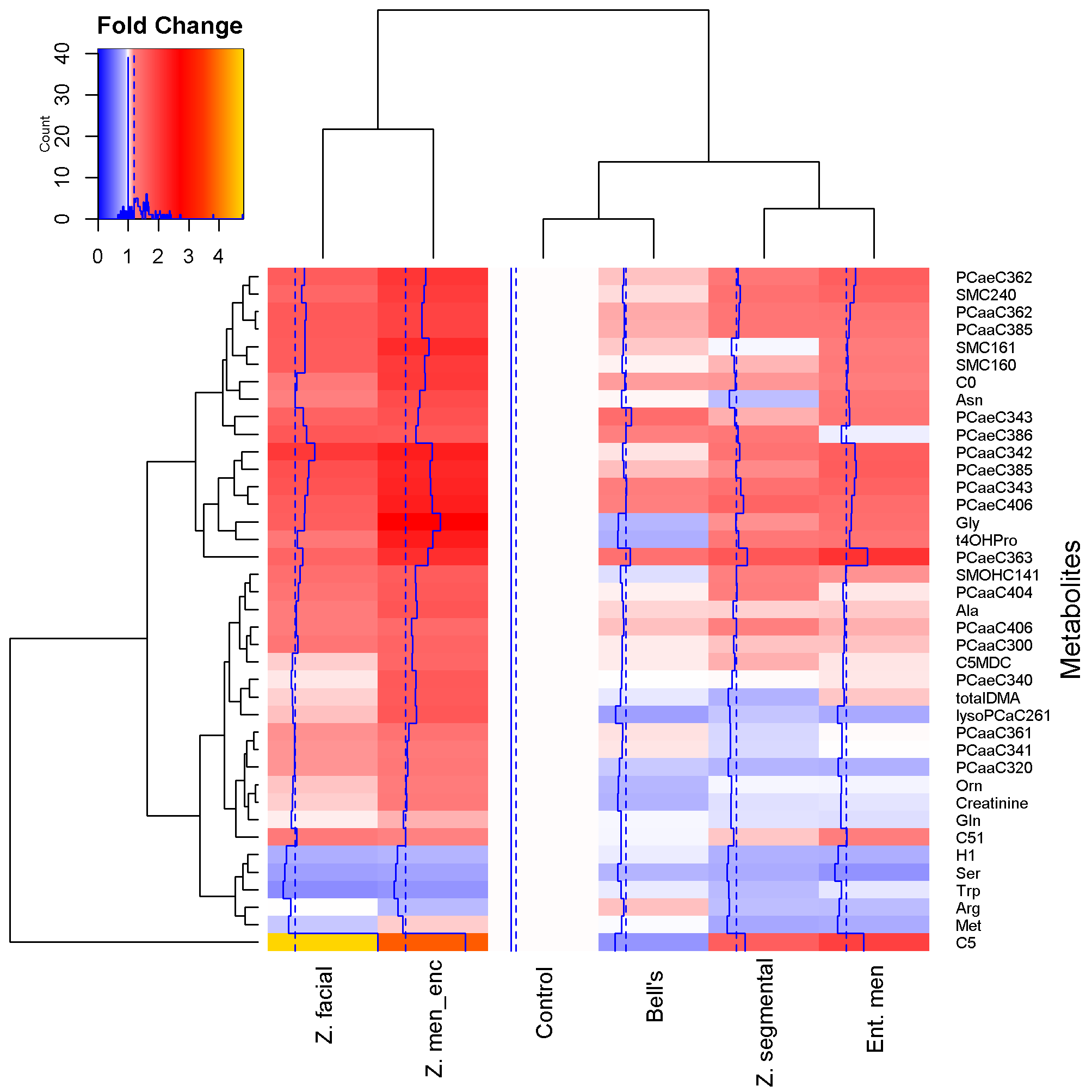

Supplement: Additional file 1: Figure S1. — Biclustering analysis based on differentially abundant metabolites. Fold changes (ratio of mean concentrations in each group relative to control) of metabolites with significant across-group differences (uncorrected Kruskal-Wallis P value < 0.05, n = 39) were used as input using the R function gplots::heatmap.2 (www.r-project.org, Authors: Andy Liaw; revised by R. Gentleman, M. Maechler, W. Huber, G. Warnes). Between-group relationships support those identified in the nonmetric MDS analysis (Fig. 2). The greatest concentration changes are evident in Z. meningoencephalitis. Some co-regulation of metabolites is evident in the dendrogram, in particular clustering of five almost exclusively downregulated metabolites (including Arg, Trp and Ser, and the sum of hexoses (H1), all of which correlated negatively with CSF leukocyte count, see Fig. 5). The apparent upregulation of C5 in the Z. facial group was due to four patients with high concentrations of unknown significance. (TIFF 337 kb) [file 12974_2017_1041_MOESM1_ESM.tiff]
